# Calculated Tumor-Associated Neutrophils Are Associated with the Tumor—Stroma Ratio and Predict a Poor Prognosis in Advanced Gastric Cancer

**DOI:** 10.3390/biomedicines10030708

**Published:** 2022-03-18

**Authors:** Eun Young Kim, Jamshid Abdul-Ghafar, Yosep Chong, Kwangil Yim

**Affiliations:** 1 Department of Surgery, Uijeongbu St. Mary Hospital, College of Medicine, The Catholic University of Korea, Seoul 06591, Korea; snuvml@catholic.ac.kr; 2 Department of Hospital Pathology, Uijeongbu St. Mary Hospital, College of Medicine, The Catholic University of Korea, Seoul 06591, Korea; jamshid@catholic.ac.kr (J.A.-G.); ychong@catholic.ac.kr (Y.C.)

**Keywords:** tumor-associated neutrophils, tumor—stroma ratio, prognostic factor, gastric cancer

## Abstract

The tumor-associated neutrophils (TANs) value and tumor—stroma ratio (TSR) are promising prognostic parameters in the tumor microenvironment. We aimed to evaluate the prognostic role and relationship of TANs and TSR in gastric cancer. Our study comprised 157 patients who underwent gastrectomy for advanced gastric cancer. TANs were assessed by immunohistochemical staining (CD15 and CD66b) and were analyzed with an image analyzer. TANs have been known to have different functional subpopulations of N1 (anti-tumor) and N2 (pro-tumor). We developed “calculated TANs with pro-tumor function (cN2; CD15 minus CD66b)”. The TSR was evaluated using hematoxylin and eosin staining. High-grade CD15-positive, cN2 in the tumor center, and TSR were significantly related to poor disease-free survival (DFS). TSR and cN2 were independent prognostic factors for DFS (hazard ratio (HR) = 2.614; *p* = 0.001, HR = 3.976; *p* = 0.002) and cN2 in the tumor center showed a positive correlation with TSR (R = 0.179, *p* = 0.025). While CD66b stained both N1 and N2, CD15 detected most of N2. Combining both markers revealed a novel cN2, which was an independent marker of poor prognosis. The transformation from N1 to N2 predominantly occurred in the tumor center, and was associated with TSR.

## 1. Introduction

Recently, tailored precision treatment such as immunotherapy has emerged as a novel strategy in gastric cancer treatment [1]. The investigation and characterization of the tumor microenvironment (TME) is a fundamental aspect of selecting personalized immunotherapeutic approaches, because TME significantly influences the therapeutic response and clinical outcome [2,3,4]. TME is a complex system composed of various cell types including tumor cells, endothelial cells, stromal cells (cancer-associated fibroblasts (CAFs)), immune cells (macrophages, lymphocytes, natural killer (NK) cells, and neutrophils), and extracellular components (cytokines, growth factors, hormones, and extracellular matrix) [2,4,5,6,7].

Tumor-associated neutrophils (TANs) represent a predominant proportion of the immune infiltrate in a variety of cancer types, including lung cancer, gastric cancers, colorectal cancer, hepatocellular carcinoma, and head and neck cancer [4]. TANs have been implicated in cancer initiation and progression, and have been identified as potential prognostic markers of various cancers [4,8,9,10]. However, previous studies that have explored the associations between TANs and gastric cancer outcomes have revealed heterogeneous results, as there are rare studies supporting both pro-tumor and anti-tumor effects of these cells in gastric cancer [11,12,13,14,15,16]. These diverse effects of TANs may come from neutrophil polarization (N1: anti-tumor function; N2: pro-tumor function) toward diametrically opposed phenotypes in response to several signals in the TME [17,18]. These studies revealed the multifaceted functional roles of TANs in different tumors, as well as in the different stages of the same tumor, although the precise mechanisms underlying TAN functions are still obscure [17,18,19].

The growing interest in TME research has also revealed the tumor—stroma ratio (TSR) (which is based on the proportions of stroma to tumor area) to be a novel potential prognostic factor in cancers [20]. Several recent reports have focused on the association between TSR and CAFs [5,20,21]. Recent studies have also investigated the interaction between CAFs and tumor-infiltrating cells, specifically TANs, within the tumor immune microenvironment, a relationship that has been identified as another key factor in promoting tumor progression [6,22,23,24]. In this process, CAFs may be able to induce the polarization of TANs to an N2 phenotype by modulating tumor cells [6]. However, the results have been limited and there have been few studies in gastric cancer [16].

In this study, we aimed to evaluate the prognostic role of TANs and their association with clinicopathological features. We also investigated the relationship between TANs and TSR in gastric cancer after gastrectomy.

## 2. Materials and Methods

### 2.1. Patients and Clinicopathological Data

A total of 157 patients with advanced gastric cancer, who underwent a gastrectomy at Uijeongbu St. Mary’s Hospital between January 2011 and December 2017, were enrolled in this study. The cohort was comprised of 150 patients who had curative surgery and 7 patients who had palliative surgery. The clinicopathological parameters were evaluated retrospectively from electronic medical records and included disease-free survival (DFS), overall survival (OS), cancer-specific survival (CSS), age, sex, size, tumor differentiation, and tumor node metastasis (TNM) stage (according to eighth TNM staging manual published by the American Joint Committee on Cancer) [25], as well as lymphatic, venous, and perineural invasion. Informed consent was obtained from all subjects involved in the study. This study was approved by the Institutional Review Board of the College of Medicine at the Catholic University of Korea (UC21SISI0011).

### 2.2. Tissue Microarray and Immunohistochemistry

**Figure 1:**
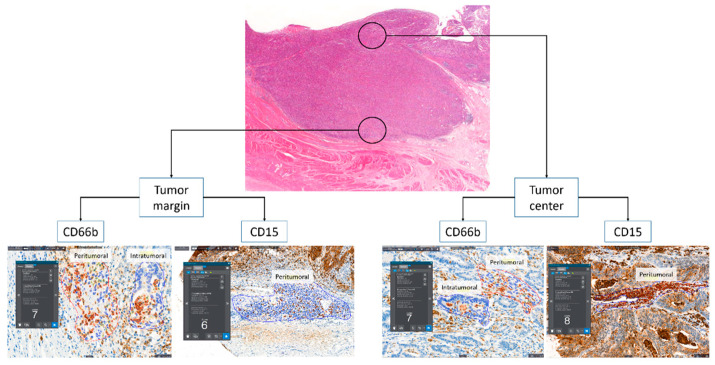
The schematic description of tumor compartments and tumor microenvironmental areas, and representative images of counting the number of neutrophils with an image analyzer.

Tissue microarrays were constructed for immunohistochemistry (IHC). Two tissue cores (3 mm) were obtained from two representative paraffin block-embedded tumor compartments (at the tumor margin and center; Figure 1). IHC staining was performed on an automated Ventana Benchmark XT platform (Roche Diagnostics, Basel, Switzerland) using monoclonal antibodies against CD15 (FDA-approved Ventana PATHWAY, MMA clone) and CD66b (1:100, 555723 [G10F5], BD biosciences, Franklin Lakes, NJ, USA), and the Ventana ultraVIEW DAB Detection Kit (Roche Diagnostics, Basel, Switzerland).

### 2.3. Image Analysis of Immunohistochemistry

IHC-stained neutrophils were counted using an image analyzer (Quant Center, 3DHISTECH, Budapest, Hungary). We selected hotspots with the most active neutrophils in the two representative tumor microenvironment areas (peritumoral and intratumoral) from the CD66b analysis (Figure 1). As the CD15 antibody stained both tumor cells and neutrophils, only peritumoral neutrophils could be analyzed (Figure 1). We calculated the average number of neutrophils per area, from areas of at least 0.1 mm^2^.

### 2.4. Tumor—Stroma Ratio (TSR)

TSR was assessed in hematoxylin and eosin (HE) stained slides at the invasive front to evaluate CAFs [5,26]. Three expert pathologists (J.A.G., Y.C. and K.Y.) independently examined each tumor section. When an ulcer was found, we excluded that section to avoid misinterpreting fibroblasts associated with ulcer-induced fibrosis as CAFs.

### 2.5. Statistical Analysis

The Chi-squared (χ^2^) test and Fisher’s exact test were used to compare low- and high-grade calculated N2 (cN2) and TSR data with clinicopathological factors. Continuous data were converted to categorical variables using cutoff values, where the sum of the sensitivity and specificity was maximized for the prediction of DFS using a time-dependent receiver operating characteristic (ROC) curve. For multivariate cox regression analyses, we used the variables that were significantly associated with DFS in a Kaplan—Meier curve analysis by log-rank tests. To minimize overfitting [27], we used lymphovascular perineural invasion (LVPI) as a parameter, which is calculated by combining lymphatic invasion, venous invasion, and perineural invasion instead of using each factor as an independent variable. Age, TNM stage, tumor size, and LVPI were used as compounding factors, to which cN2 and TSR were added one by one. Two-sided *p* values < 0.05 were considered statistically significant. All of the analyses were performed using SPSS software (version 20.0; IBM, Armonk, NY, USA) and R statistical programming (version 3.4.1; http//www.r-project.org, accessed on 7 March 2022).

## 3. Results

### 3.1. Prognostic Value of TANs and Establishment of cN2

Our analyses indicated that neutrophils detected by the CD66b antibody were not associated with DFS, OS, or CSS. However, CD66b-postive neutrophils had a tendency to be associated with a good prognosis when located in the tumor margin and peritumoral area of the tumor center, but were correlated with a poor prognosis when the cells were present in the intratumoral area of the tumor center, although these results were not statistically significant (Table 1 and Supplementary Figure S1). The number of neutrophils stained by the CD15 antibody were correlated with poor DFS, OS, and CSS when the cells were located in the tumor margin and center (*p* = 0.006 and *p* = 0.002 in DFS, *p* = 0.001 and *p* = 0.009 in OS, and *p* = 0.014 and *p* = 0.011 in CSS; Table 1 and Supplementary Figure S1).

By combining the values from both CD15- and CD66b-positive staining, we developed a measurement that we call “calculated TANs with pro-tumor function (cN2)”. The cN2 value is defined as the number of CD66b-postive cells subtracted from the number of CD15-positive cells.

### 3.2. Clinicopathologic Characteristics Associated with cN2 and TSR in Patients with Advanced Gastric Cancer

Recurrence occurred in 62 patients (39.5%), death in 100 (63.7%), and cancer-specific death in 63 (40.1%) out of 157 patients. Recurrence, death, cancer-specific death, advanced pT stage, and lymphatic and perineural invasion were significantly associated with high-grade cN2. High-grade TSR had a significant relationship with recurrence, death, cancer-specific death, advanced pT stage, pN stage, pM stage, and pTNM stage, as well as lymphatic, venous, and perineural invasion (Table 2).

### 3.3. Prognostic Value of cN2 and TSR in Patients with Advanced Gastric Cancer

**Figure 2:**
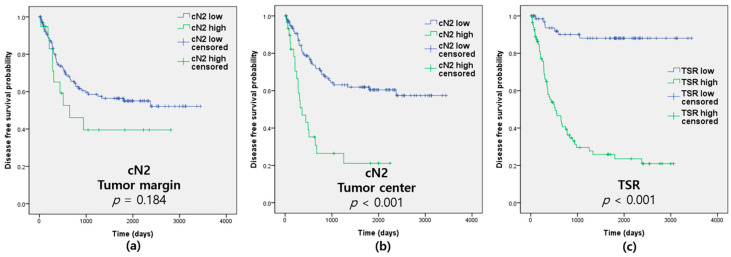
Disease-free survival according to calculated tumor-associated neutrophils for pro-tumor function (cN2) and tumor—stromal ratio (TSR). (**a**) cN2 at the tumor margin; (**b**) cN2 at the tumor center; (**c**) TSR.

The cN2 value was associated with a poor DFS for cells located in the tumor center (*p* < 0.001) but not in the tumor margin (*p* = 0.184; Figure 2 and Table 1). High-grade TSR was revealed to be a marker of poor DFS, OS, and CSS (*p* < 0.001, *p* < 0.001, and *p* < 0.001; Figure 2 and Table 1). pTNM stage, lymphatic, venous and perineural invasion, tumor size, and neutrophils detected by CD15 were also significantly associated with prognosis (Table 3).

### 3.4. Multivariate Cox Proportional Hazards Model and Correlation between TANs and TSR

High-grade cN2 was an independent predictor of poor DFS for cells located in the tumor center (*p* = 0.001), but not in the tumor margin (*p* = 0.860). High-grade TSR was also an independent marker of poor prognosis (*p* = 0.002; Table 3). Neutrophils detected by CD66b (data not shown) and CD15 individually were not independent prognostic factors (Table 3). TSR was positively correlated with the cN2 value in the tumor center (R = 0.179, *p* = 0.025) and was negatively correlated with CD66b-positive neutrophils in the intratumoral area at the tumor margin (R = −0.185, *p* = 0.020; Table 4).

## 4. Discussion

Our study demonstrated that the cN2 value is an independent marker of a poor prognosis for DFS and OS (Table 3). To the best of our knowledge, this is the first study to reveal that different antibodies can detect distinct neutrophils with opposite prognostic effects. Furthermore, we also established a novel marker, cN2, which is an independent predictor of a poor prognosis.

The role of TANs in the TME remains controversial, with evidence for both pro- and anti-tumor roles [28]. In several studies, high densities of TANs were independently associated with an unfavorable prognosis [8,12,16,29,30,31]. However, there have also been studies that have reported TANs as a favorable prognostic factor or demonstrated no relationship between TANs and prognosis [8,11,13,14,15,32]. Even in the same cancer, the prognostic trends have not been consistent [8]. The dual functions of TANs originate from the plasticity of neutrophils in response to a variety of stimuli [33,34]. TAN polarization probably exists as a spectrum of activation states, rather than only two extremes; namely the N1 or N2 categories [35]. This dual function is likely a reflection of their unexpected plasticity in response to environmental cues, such as transforming growth factor β (TGF-β) and interferon β (IFN-β) [17].

The markers used to identify TANs (such as CD66b, CD15, myeloperoxidase, and cell morphology by HE staining) may explain these discrepancies, at least partly [11,12,13,14,15,16,29,30,31,32,36,37]. The expression of these markers in neutrophils may vary, resulting in different prognostic effects [9]. CD15 is expressed in neutrophils, eosinophils, some monocytes, and occasionally in tumor cells [38]. It is also a differentiation marker found on all neutrophil subpopulations [39]. CD66b immunoreactivity is found on both neutrophils and eosinophils, and is recognized as a granulocyte activation marker and neutrophil lineage marker [39,40]. In the present study, we evaluated both CD66b- and CD15-postive TANs simultaneously, and revealed that they identified TANs with the opposite prognostic effects, using different immunostaining markers.

We revealed that high-grade CD66b-positive TANs have different prognostic effects according to the tumor compartments or microenvironments. In the majority of previously reported studies, the average numbers of CD66b-postive TANs without differentiating by compartment or microenvironments were analyzed, and variously described a high density of CD66b-postive TANs as having good [13,14], poor [16,31], or not significant [32] prognostic factors. In the present study, we revealed that a high density of CD66b-positive TANs in the intratumoral area of the tumor central compartment was associated with a trend toward poor survival; however, the same cells located in other areas showed good survival trends, although they were not statistically significant (data not shown). These results suggest that if previous researchers focused their analyses on the intratumoral area of the central compartment, they would have found CD66-postive TANs to be a poor prognostic factor; while if they evaluated other areas, they would have identified CD66-postive TANs to be a good prognostic factor. Therefore, it is necessary to evaluate TANs within different tumor compartments and microenvironments, at least in gastric cancer.

Li et al. and Liu et al. evaluated the CD66b-positive TANs only with the tumor compartment, and not the tumor microenvironment areas [30,36]. When Li et al. divided their tumor analyses based on the invasive margin and tumor center, their multivariate analyses revealed that a high level of CD66b-postive TANs in the invasive margin was an independent marker of poor prognosis, but not when the cells were located in the tumor center [30]. In addition, Liu et al. evaluated TANs in the tumor center and the invasive border of the tumor compared to normal tissues [36]. In the univariate analysis, they showed that high-grade CD66b-positive TANs were associated with a poor prognosis in normal tissue and the tumor center, but a good prognosis when cells were found at the invasive border. They also established a ratio of CD66b-postive TANs between the tumor center and the invasive border, and showed that it was an independent marker of a poor prognosis [36]. In the present study, high-grade CD66b-postive TANs (combining peritumoral and intratumoral) were a significantly good survival marker in the tumor margin (*p* = 0.044), but not significant in the tumor center (*p* = 0.011), with trends toward poor survival in the univariate analysis, while the results were not significant in the multivariate analysis in both tumor regions (margin: *p* = 0.066, center: *p* = 0.850; data not shown). These findings were similar to those reported by Liu et al. [36], but did not match those from Li et al. [30]. The results of the ratio between the tumor center and the margin were similar only in the univariate analysis (*p* = 0.006), but there was no significance in the multivariate analysis (*p* = 0.538) (data not shown). One reason that our results differ from those of Li et al. may be that we analyzed different populations or employed different methods of screening. In each slide, we only selected the most active hotspots in the intratumoral and peritumoral areas, and used an automated analyzer to obtain more objective results.

In contrast to the CD66 expression, CD15-positive TANs displayed poor prognostic effects in all previous studies that analyzed the mean number of TANs without differentiating regions or areas [12,29,31]. Similarly, we revealed that high-grade CD15-positive TANs were a poor prognostic marker. Furthermore, because CD15 also stained the tumor cells, we could only evaluate TANs in the peritumoral area.

In the present study, we established the cN2 measurement by focusing on the tendency for CD15 to stain more N2 cells compared to CD66b, which stained both N1 and N2 cells (but stained more N1 cells). As a result, cN2 is a poor prognostic factor in the tumor center, and CD66b and CD15 alone could not predict prognosis, as only the univariate analysis suggested they might be significant markers. In contrast, cN2 was shown to be an independent prognostic factor in the tumor center. We also revealed that the transformation from N1 to N2 predominantly occurred in the tumor center, as suggested by Mishalian et al. [41]. These authors reported that neutrophils remain predominantly located at the edges of the tumor and have an N1 phenotype at early stages. However, as the tumor progresses, neutrophils are often found deeper within the tumor and possess an N2 phenotype, which supports tumor growth [41].

We demonstrate that cN2 is closely correlated with clinicopathological factors such as T stage, and lymphatic and perineural invasion. In contrast, Wang et al. suggested that TANs may help to estimate lymph node metastasis in early gastric cancer [37]. Abe et al. revealed that in Epstein–Barr virus-associated gastric cancer, a high density of CD66b-positive cells is associated with intestinal-type histology and a low frequency of lymph node metastasis [32]. In the present study, there was no association with pN or with intestinal type. We presume that this is because the previous study evaluated early gastric cancer, and they measured TANs primarily by HE staining with the aid of myeloperoxidase IHC. In this study, we established the cN2 measurement using CD66b and CD15 IHC analyses that were evaluated with an image analyzer.

In addition, we found that TSR is an independent predictor of a poor prognosis. In particular, fibrosis in the tumor center also appeared to play an important role in the transformation of neutrophils from an N1 to N2 phenotype. Accordingly, as stroma-rich tumors have more CAFs, they could reasonably benefit from greater support of tumor growth [21]. The cancer stroma, as exemplified by CAFs, plays critical roles in cancer invasion and metastasis [5]. TGF-β by itself stimulates an N2 phenotype and inhibits N1 phenotypic polarization in neutrophils, whereas IFN-β stimulates N1 while inhibiting N2 polarization [10,19,42]. CAF-mediated TGF-β signaling redirects TAN differentiation toward the N2 phenotype. Conversely, a TGF-β blockade attenuates tumor growth via TAN polarization to an anti-tumor N1 phenotype, thereby providing additive strategies for cancer therapies [43].

Our study included a relatively small number of patients analyzed by retrospective data collection. Further prospective research on a larger scale is required to confirm our findings. However, as we collected and analyzed all of the gastric cancer cases in one institution over the course of six years, our study cohort might well reflect the results of real-world practice. In addition, interobserver variations may exist in the interpretation of TSR. To overcome this weakness, in the present study, TSR was evaluated independently by three expert pathologists (J.A.G., Y.C. and K.Y.) Finally, while we suggest that the cN2 value represents N2 neutrophils, we do not have definitive evidence that cN2 only detects N2 cells. Further studies are required to confirm that cN2 represents only the N2 population.

## 5. Conclusions

CD66b stained both the N1 and N2 populations of neutrophils; however, CD15 detected more N2 cells. By combining both markers, we outline a novel measurement, cN2 (CD15 minus CD66), that is an independent marker of a poor prognosis. The transformation from N1 to N2 occurred predominantly in the tumor center and was associated with the TSR.

## Figures and Tables

**Table 1:** The prognostic effects of the clinicopathological factors, tumor–associated neutrophils, and tumor—stroma ratio using the Kaplan—Meier curve analysis.

Factors			Cutoff Values	DFS	OS	CSS
				*p* Value	*p* Value	*p* Value
Age			≤ vs. >72 years old	0.268	<0.001	0.131
Sex			Male vs. female	0.378	0.984	0.641
pT			T2 + T3 vs. T4	<0.001	<0.001	<0.001
pN			N0 vs. N1 + N2 + N3	<0.001	<0.001	<0.001
pM			M0 vs. M1	0.069	0.001	<0.001
pTNM			I + II vs. III + IV	<0.001	<0.001	<0.001
Lauren classification			Intestinal vs. Others	0.146	0.519	0.127
Tumor differentiation			Well + moderately vs. poorly	0.130	0.171	0.054
Lymphatic invasion			Absent vs. present	<0.001	<0.001	<0.001
Venous invasion			Absent vs. present	0.003	0.096	0.015
Perineural invasion			Absent vs. present	<0.001	<0.001	<0.001
CD66b	Margin	Peritumoral	≤ vs. >54.81/mm^2^	0.104	0.219	0.081
		Intratumoral	≤ vs. >30.6/mm^2^	0.167	0.048	0.047
	Center	Peritumoral	≤ vs. >4.8/mm^2^	0.053	0.169	0.097
		Intratumoral	≤ vs. >6.5/mm^2^	0.124	0.372	0.171
CD15	Margin		≤ vs. >2473.3/mm^2^	0.006	0.001	0.014
	Center		≤ vs. >536.1/mm^2^	0.002	0.009	0.011
cN2	Margin		≤ vs. >949.3/mm^2^	0.184	0.038	0.044
	Center		≤ vs. >954.6/mm^2^	<0.001	0.002	0.002
TSR			≤ vs. >40%	<0.001	<0.001	<0.001

DFS, disease free survival; OS, overall survival; CSS, cancer specific survival; cN2, calculated tumor-associated neutrophils for pro-tumor function; TSR, tumor—stromal ratio.

**Table 2:** Clinicopathological characteristics according to the calculated tumor-associated neutrophils for pro-tumor function and tumor—stromal ratio.

Factors	cN2 of Tumor Center		*p* Value	TSR		*p* Value
	Low-Grade (≤954.6/mm^2^) n = 126 (%)	High-Grade (>954.6 /mm^2^) n = 31 (%)		Low-Grade (≤40%) n = 72 (%)	High-Grade (>40%) n = 85 (%)	
Age (years old)			0.681			0.857
≤72	58 (81.7)	13 (18.3)		32 (45.1)	39 (54.9)	
>72	68 (79.1)	18 (20.9)		40 (46.5)	46 (53.5)	
Sex			0.167			0.898
Male	93 (83.0)	19 (17.0)		51 (45.5)	61 (54.5)	
Female	33 (73.3)	12 (26.7)		21 (46.7)	24 (53.3)	
pT			0.001			0.000
T2 + T3	88 (88.0)	12 (12.0)		60 (60.0)	40 (40.0)	
T4	38 (66.7)	19 (33.3)		12 (21.1)	45 (78.9)	
pN			0.089			<0.001
N0	49 (87.5)	7 (12.5)		41 (73.2)	15 (26.8)	
N1 + N2 + N3	77 (76.2)	24 (23.8)		31 (30.7)	70 (69.3)	
pM			0.591			0.013
M0	114 (79.7)	29 (20.3)		70 (49.0)	73 (51.0)	
M1	12 (85.7)	2 (14.3)		2 (14.3)	12 (85.7)	
pTNM			0.260			<0.001
I + II	63 (84.0)	12 (16.0)		54 (72.0)	21 (28.0)	
III + IV	63 (76.8)	19 (23.2)		18 (22.0)	64 (78.0)	
Lauren classification			0.577			0.764
Intestinal	68 (81.9)	15 (18.1)		39 (47.0)	44 (53.0)	
Others	58 (78.4)	16 (21.6)		33 (44.6)	41 (55.4)	
Differentiation			0.240			<0.001
Well + Moderate	51 (85.0)	9 (15.0)		30 (50.0)	30 (50.0)	
Poorly	75 (77.3)	22 (22.7)		42 (43.3)	55 (56.7)	
Lymphatic invasion			0.014			<0.001
Absent	59 (89.4)	7 (10.6)		49 (74.2)	17 (25.8)	
Present	67 (73.6)	24 (26.4)		23 (25.3)	68 (74.7)	
Venous invasion			0.223			0.001
Absent	115 (81.6)	26 (18.4)		71 (50.4)	70 (49.6)	
Present	11 (68.8)	5 (31.3)		1 (6.3)	15 (93.8)	
Perineural invasion			<0.001			<0.001
Absent	85 (89.5)	10 (10.5)		60 (63.2)	35 (36.8)	
Present	41 (66.1)	21 (33.9)		12 (19.4)	50 (80.6)	
Tumor size (mm)			0.074			0.079
≤55.0	68 (86.1)	11 (13.9)		42 (53.2)	37 (46.8)	
>55.0	58 (74.4)	20 (25.6)		30 (38.5)	48 (61.5)	
Overall death			0.009			<0.001
Alive	52 (91.2)	5 (8.8)		45 (78.9)	12 (21.1)	
Death	74 (74.0)	26 (26.0)		27 (27.0)	73 (73.0)	
Recurrence			0.001			<0.001
Absent	84 (88.4)	11 (11.6)		65 (68.4)	30 (31.6)	
Present	42 (67.7)	20 (32.3)		7 (11.3)	55 (88.7)	
Cancer-specific death			0.023			<0.001
Alive	81 (86.2)	13 (13.8)		65 (69.1)	29 (30.9)	
Death	45 (71.4)	18 (28.6)		7 (11.1)	56 (88.9)	

cN2, calculated tumor-associated neutrophils for pro-tumor function; TSR, tumor—stromal ratio.

**Table 3:** Univariate and multivariate analyses of factors associated with disease-free survival of advanced gastric cancer.

		Univariate Analysis		Multivariate Analysis	
		HR (95% CI)	*p* Value	Adjusted HR (95% CI)	*p* Value
Age (>72 years)		1.326 (0.803–2.190)	0.270		
pTNM (III + IV)		13.386 (6.031–29.708)	<0.001		
LVPI (Present)		11.050 (4.001–30.521)	<0.001		
Tumor size (>55.0 mm)		2.761 (1.627–4.687)	<0.001		
CD15	Margin (>2473.3/mm^2^) *	2.269 (1.149–4.484)	0.018	1.539 (0.746–3.174)	0.243
	Center (>536.1/mm^2^) *	2.393 (1.287–4.448)	0.006	1.106 (0.597–2.050)	0.749
cN2	Margin (>949.3/mm^2^) *	1.576 (0.800–3.106)	0.188	1.066 (0.521–2.181)	0.860
	Center (>954.6/mm^2^) *	3.241 (1.888–5.566)	<0.001	2.614 (1.436–4.671)	0.001
TSR (>40%) *		10.768 (4.877–23.775)	<0.001	3.976 (1.643–9.620)	0.002

HR, hazard ratio; CI, confidence interval; LVPI, lymphovascular and perineural invasion; cN2, calculated tumor-associated neutrophils for pro-tumor function; TSR, tumor—stromal ratio. * Adjusted for age, pTNM, lymphovascular and perineural invasion, and tumor size.

**Table 4:** Correlation of variable tumor associated neutrophils, calculated tumor-associated neutrophils for pro-tumor function, and tumor—stromal ratio.

Factors			Pearson’s Coefficient	*p* Value
CD66b	Margin	Peritumoral	−0.095	0.235
		Intratumoral	−0.185	0.020
	Center	Peritumoral	−0.085	0.288
		Intratumoral	−0.067	0.403
CD15	Margin	Peritumoral	0.035	0.660
	Center	Peritumoral	0.133	0.096
cN2	Margin	Peritumoral	0.088	0.275
	Center	Peritumoral	0.179	0.025

cN2, calculated tumor-associated neutrophils for pro-tumor function.

## Data Availability

Data can be made available upon reasonable request.

## Supplementary Materials

The following supporting information can be downloaded at: https://www.mdpi.com/article/10.3390/biomedicines10030708/s1, Supplementary Figure S1: Disease-free survival according to tumor-associated neutrophils detected with CD66b and CD15.
