# Supplementary material for: Calculated Tumor-Associated Neutrophils Are Associated with the Tumor—Stroma Ratio and Predict a Poor Prognosis in Advanced Gastric Cancer
Source: Biomedicines. 2022 Mar 18;10(3):708. doi: 10.3390/biomedicines10030708 (PMC8945075; doi:10.3390/biomedicines10030708)
Supplement: Supplementary file 1 [file biomedicines-10-00708-s001.zip › biomedicines-1638443-supplementary-3.21.pdf]

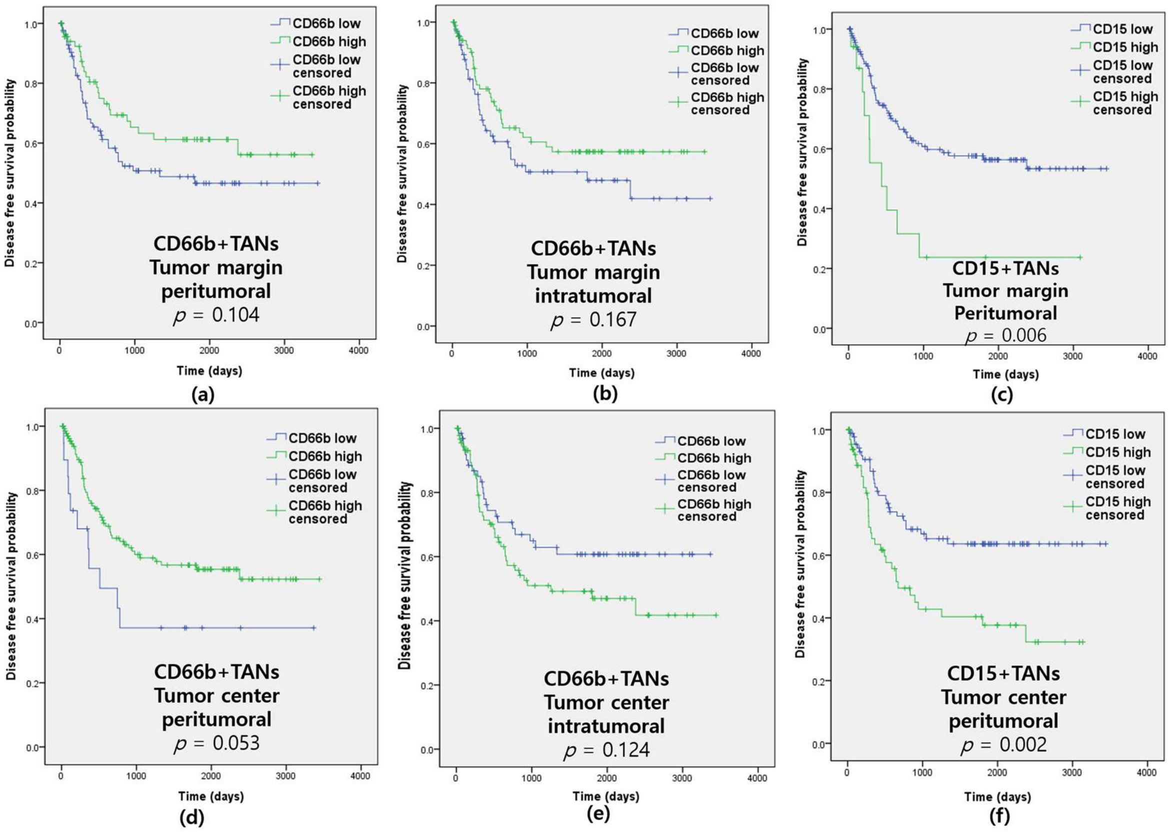

Supplementary Figure S1: Disease-free survival according to tumor-associated neutrophils detected with CD66b and CD15.
